# Addition of soluble fiber to standard purified diets is important for gut morphology in mice

**DOI:** 10.1038/s41598-023-46331-5

**Published:** 2023-11-07

**Authors:** Marietta von Süßkind-Schwendi, Andreas Dötsch, Vivien Haberland, Paola Ferrario, Ralf Krüger, Sandrine Louis, Maik Döring, Daniela Graf

**Affiliations:** 1 Department of Physiology and Biochemistry of Nutrition, Max Rubner-Institut (MRI)-Federal Research Institute of Nutrition and Food, Haid-und-Neu-Straße 9, 76131 Karlsruhe, Germany; 2 National Reference Centre for Authentic Food, Max Rubner-Institut (MRI)-Federal Research Institute of Nutrition and Food, E.-C.-Baumann-Straße 20, 95326 Kulmbach, Germany

**Keywords:** Biochemistry, Biological techniques, Physiology, Medical research

## Abstract

Purified diets (PD) increase standardization and repeatability in rodent studies but lead to differences in the phenotype of animals compared to grain-based “chow” diets. PD contain less fiber and are often devoid of soluble fiber, which can impact gut health. Thus, the aim of the present study was to modify the PD AIN93G by addition of soluble fiber, to promote more natural gut development as seen with chow diets. One hundred twenty male C57BL/6J mice were fed over 12 weeks either a chow diet, AIN93G or one of three modified AIN93G with increased fiber content and different ratios of soluble fiber to cellulose. Gut health was assessed through histological and immunohistochemical parameters and gut barrier gene expression. Gut microbiota composition was analyzed and its activity characterized through short chain fatty acid (SCFA) quantification. Feeding AIN93G led to tissue atrophy, a less diverse microbiota and a lower production of SCFA compared to chow diet. The addition of soluble fiber mitigated these effects, leading to intermediate colon and caecum crypt lengths and microbiota composition compared to both control diets. In conclusion, the addition of soluble fibers in PDs seems essential for gut morphology as well as a diverse and functional gut microbiome.

## Introduction

Choosing a good control diet is pivotal for the outcome and interpretation of study results obtained in animal studies^1–3^. There are two different kind of diets that are widely used: (a) grain-based “standard chow” diets and (b) purified diets, e.g., AIN93G.

“Standard chow” diets are a complex mixture of different grains. Each producer has its own secret formulation for these chow diets and as they consist of non-purified grains, there are batch-to-batch differences in (micro) nutrient composition^4,5^. Therefore, it is not clear, which nutrients are contained in a specific chow diet used for a certain study. For example, the concentrations of dietary fiber, which is well known to impact health, have been reported to vary from batch to batch ranging from 3 to 7% for soluble fiber in three different lots^6^.

Furthermore, these chow diets are known to contain non-nutrients (e.g. phytoestrogens), heavy metals (e.g. lead) and contaminants such as pesticides and mycotoxins^4,5^. It has been reported that occurrence of these unwanted substances in varying concentrations can impact study outcomes and data interpretation. One of the best studied compounds in this regard are phytoestrogens. As reviewed by Pellizzon and Ricci^4^ concentrations of phytoestrogens in chow diets range from < 100 mg/kg up to 800 mg/kg. And even for the same diet up to sixfold differences between lots have been reported. Phytoestrogens can bind to the estrogen receptors and thus have an impact on various aspects of animal physiology, such as maturation or carcinogenesis, which are affected by the use of chow diets^4^. Because of this, grain-based chow diets are not a satisfactory feeding for many studies^5^.

In contrast to chow diets purified diets have several advantages. Using a purified diet enables the researcher to report exactly what diet was used and which ingredients it consists of, as formulations are available^4^. It also allows for better repeatability, as the diets contain refined ingredients and can be reproduced easily^5^. This is especially important for nutritional intervention studies, considering that diets can easily be adapted, meaning single nutrients can be added or removed^4^. Thus, for high quality, well controlled, repeatable experiments, particularly for nutrition studies, purified diets are recommended.

However, it has been reported that mice/rats receiving purified diets differ in their phenotype compared to mice/rats receiving chow diets. A reduction of colon lengths and reduced caecum weight as well as an increased fat pad and body weight have been reported among animals being fed a purified diet compared to chow diet^7^. As wild-living animals do not have access to purified diets, chow diets are probably more representable.

The main differences between chow and purified diets include dietary fiber level and type^8^. In the last couple of years, research interest in dietary fiber has been renewed, due to its huge impact on gut microbiota and gut health^9^. In particular, soluble dietary fibers serve as energy substrate for the gut microbiota and are metabolized to short-chain fatty acids (SCFA), which in turn can impact (gut) health of the host^10^.

Chow diets do not only contain more dietary fiber compared to purified diets (usually 15–20% compared to 5%), but they contain a complex mixture of soluble and insoluble dietary fibers, whereas standard purified diets such as AIN93 and AIN76 contain only insoluble cellulose, which is poorly metabolized by murine and human gut microbiota^11^. It has been suggested that this might not be adequate for the development of a healthy gut^8^.

Therefore, the aim of the present study was to modify the standard purified diet AIN93G by increasing the total fiber content and adding soluble fibers, to enhance and mimic gut development as seen with a chow diet. Thus, an increase in the amount of dietary fiber to 15%, which is on the lower end of the range found in chow diets, but already three times as much as contained in normal AIN93G was chosen. To avoid compromising repeatability of the purified diets, well standardized and easy to obtain soluble fibers (inulin and pectin) were used. In this study a 1:1 mixture of soluble dietary fibers (inulin and pectin) was added in different ratios with cellulose (70:30, 50:50, 30:70), to test our main hypothesis that an addition of soluble fibers in different quantities will influence differently gut development, especially colon length growth. The impact of these three modified diets abbreviated with 70S, 50S, and 30S and the two control diets chow and AIN93G on gut morphology, gut microbiota composition and production of SCFA and branched-chain fatty acids (BCFA) was investigated.

## Results

### Food/energy intake and body weight

Mice receiving chow diet had a higher food intake compared to the other diets (Table 1). Combined with the different energy densities of the diets, this led to a lower energy intake by mice fed modified AIN diets compared to mice receiving either chow diet or AIN93G (Table 1). Overall the body weight gain and final body weight of the mice were similar among the groups, except for the 50S group, which showed a reduced body weight and weight gain compared to the AIN95G group.

**Table 1:** Average food and energy intake during the study period (per mouse) and morphological parameters.

	Chow	70S	50S	30S	AIN93G
Food intake [g/day]	3.8 ± 0.2*	2.8 ± 0.1*^#^	2.7 ± 0.2*^#^	3.0 ± 0.2^#^	3.1 ± 0.2^#^
Energy intake [MJ]	4.2 ± 0.3	3.4 ± 0.2*^#^	3.3 ± 0.3*^#^	3.6 ± 0.3*^#^	4.1 ± 0.3
Final body weight [g]	27.6 ± 2.0	27.7 ± 2.1	27.0 ± 2.0*	28.1 ± 1.9	29.2 ± 1.7
Body weight gain [g]	17.7 ± 1.8	17.7 ± 2.5	16.8 ± 2.0*	18.1 ± 2.9	18.7 ± 2.0
Final body length [cm]	9.4 ± 0.3	9.4 ± 0.2	9.3 ± 0.3	9.4 ± 0.2	9.5 ± 0.2
Colon length [cm]	8.6 ± 0.6*	8.4 ± 1.0*	7.9 ± 0.9*^#^	7.9 ± 0.6*^#^	7.3 ± 0.6^#^
Caecum weight [g]	0.56 ± 0.09*	0.66 ± 0.18*^#^	0.50 ± 0.09*	0.38 ± 0.06*^#^	0.27 ± 0.04^#^

All values presented are mean ± SD.

^#^p < 0.05 Dunnett test group-wise comparison to chow.

*p < 0.05 Dunnett test group-wise comparison to AIN93G.

70S: experimental diet with fiber fraction consisting of 70% soluble fiber and 30% cellulose.

50S: experimental diet with fiber fraction consisting of 50% soluble fiber and 50% cellulose.

30S: experimental diet with fiber fraction consisting of 30% soluble fiber and 70% cellulose.

### Colon morphology is impacted by different control diets

**Figure 1:**
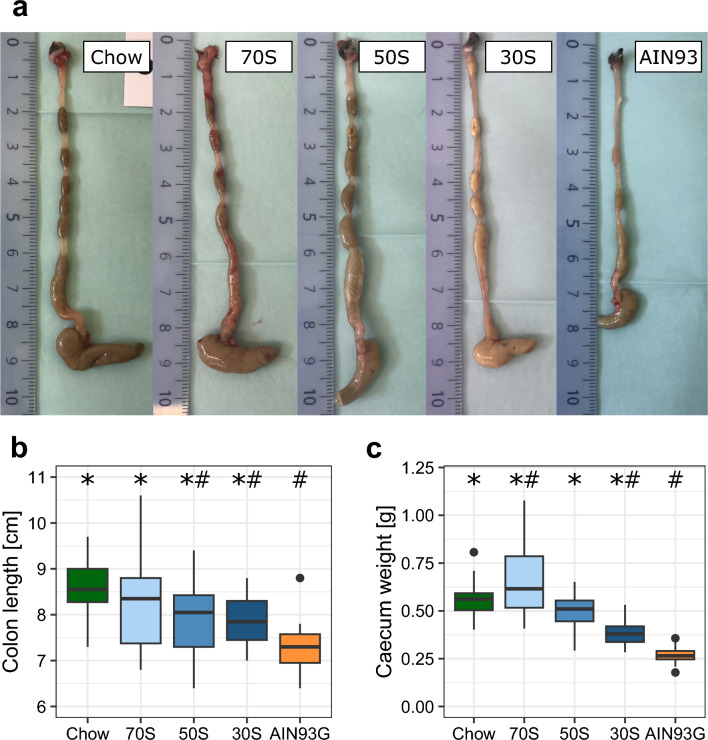
Representative pictures of colon and caecum for each group are provided in panel (**a**). The pictures were not used to measure colon lengths, they are for demonstration purpose only. Colon length (**b**) and caecum weight (**c**) of mice fed either chow diet, AIN93G or an AIN-based diet with higher fiber content and varying ratios of soluble fiber and cellulose for 12 weeks. Boxplots display median and quartiles of the data points (n = 24 per group). 70S: experimental diet with fiber fraction consisting of 70% soluble fiber and 30% cellulose; 50S: experimental diet with fiber fraction consisting of 50% soluble fiber and 50% cellulose; 30S: experimental diet with fiber fraction consisting of 30% soluble fiber and 70% cellulose. * and ^#^ indicate significant difference to AIN93G and Chow, respectively (p < 0.05, Dunnett’s Test).

**Figure 2:**
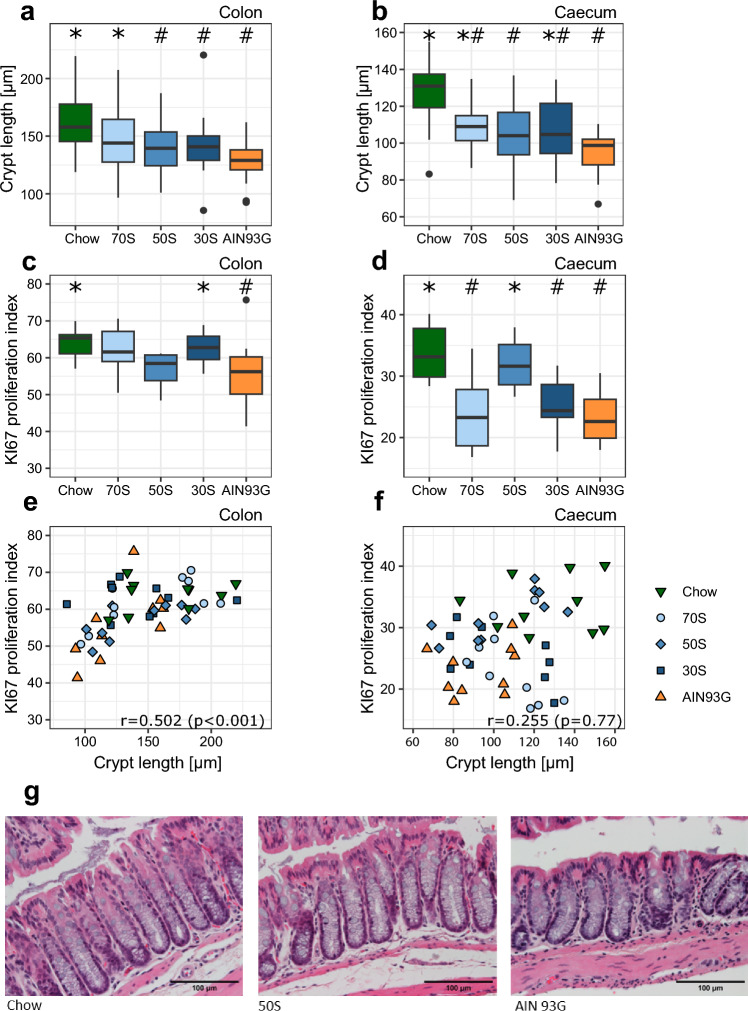
Crypt length (**a**,**b**), proliferation index (**c**,**d**) and scatter plot of crypt length and proliferation index (**e**,**f**) for colon (**a**,**c**,**e**) and caecum (**b**,**d**,**f**) of mice fed either chow diet, AIN93G or one of three AIN-based diets with varying ratios of soluble fiber to cellulose. Representative colon HE stains are provided in (**g**). Boxplots display median and quartiles of the data points. Crypt lengths were measured in 19–24 samples per group and KI67 in 10 samples per group (9 in caecum of group “30S”). In the scatter plots, each point represents an individual measurement. 70S: experimental diet with fiber fraction consisting of 70% soluble fiber and 30% cellulose; 50S: experimental diet with fiber fraction consisting of 50% soluble fiber and 50% cellulose; 30S: experimental diet with fiber fraction consisting of 30% soluble fiber and 70% cellulose. * and ^#^ indicate significant difference to AIN93G and Chow, respectively (p < 0.05, Dunnett’s Test).

Colon morphology, especially colon length was our primary study outcome. Mice receiving the AIN93G diet had the lowest average colon length of all groups (Table 1, Fig. 1a,b). Mice, receiving the modified AIN diets had colon lengths intermediate to the colon lengths in AIN93G and chow. Average colon length increased with increased content of soluble fiber. The 70S diet group showed similar colon lengths to the chow group. Similar results were found for crypt length which was reduced in AIN93G animals compared to the chow diet group and intermediate in the modified AIN diets with 50S and 30S fed animals showing shorter crypt length than chow fed animals. The 70S group showed increased crypt length compared to the AIN93G group and no significant difference to the chow diet group (Fig. 2a). To further investigate whether the increase in colon (crypt) length was associated with an increased proliferation, we assessed the KI67 proliferation index. Proliferation index and crypt length correlated moderately (Fig. 2e). The two standard control diets (chow and AIN93G) differed in proliferation index, with AIN93G showing a lower proliferation. Furthermore, 30S had an increased proliferation compared to AIN93G. However, no significant differences were observed between 70S and 50S and the two control diets (Fig. 2c).

In addition to the morphological parameters examined above, we assessed the abundance of goblet cells lining the crypts. No significant differences were observed between the three modified AIN-based diets and the two standard control diets (Supplementary Fig. S1a).

To investigate whether colon barrier function might be impacted by the differences observed, we assessed gene expression of tight junction proteins JAM-A, E-cadherin, Occludin and ZO-1. Gene expression of E-cadherin was slightly higher in chow-fed mice compared to AIN93G and 50S-fed mice, but otherwise no significant differences were observed (Table 2).

**Table 2:** Gene expression of tight junction proteins and MUC-2 in gut tissue (colon and ileum).

		Chow	70S	50S	30S	AIN93G
Colon	JAMA	1.1 ± 0.1	1.0 ± 0.2	1.1 ± 0.3	1.1 ± 0.1	1.0 ± 0.3
	E-Cadherin	1.1 ± 0.2*	0.9 ± 0.2	0.9 ± 0.2^#^	1.0 ± 0.1	0.8 ± 0.2^#^
	ZO-1	1.1 ± 0.2	0.9 ± 0.2	1.0 ± 0.2	1.0 ± 0.2	1.1 ± 0.3
	Occludin	1.0 ± 0.2	1.0 ± 0.2	1.0 ± 0.3	1.0 ± 0.1	0.9 ± 0.3
Ileum	JAMA	1.0 ± 0.3*	0.8 ± 0.3	0.7 ± 0.3	0.7 ± 0.2^#^	0.7 ± 0.4^#^
	E-Cadherin	1.0 ± 0.3	1.0 ± 0.2	0.9 ± 0.3	1.0 ± 0.2	0.9 ± 0.3
	ZO-1	1.0 ± 0.2*	0.9 ± 0.3	0.8 ± 0.3	0.8 ± 0.1	0.8 ± 0.2^#^
	Occludin	1.1 ± 0.2*	0.8 ± 0.3^#^	0.7 ± 0.2^#^	0.8 ± 0.3^#^	0.8 ± 0.3^#^
	MUC-2	1.1 ± 0.6	1.1 ± 0.4	0.8 ± 0.4	1.2 ± 0.7	0.8 ± 0.4

All values presented are mean ± SD.

^#^p < 0.05 Dunnett test group-wise comparison to chow.

*p < 0.05 Dunnett test group-wise comparison to AIN93G.

70S: experimental diet with fiber fraction consisting of 70% soluble fiber and 30% cellulose.

50S: experimental diet with fiber fraction consisting of 50% soluble fiber and 50% cellulose.

30S: experimental diet with fiber fraction consisting of 30% soluble fiber and 70% cellulose.

### Caecum morphology is impacted by different control diets

In mice the caecum is the main site where bacterial fermentation takes place and dietary fiber is metabolized to SCFA. Therefore, we also investigated the impact of our diets on caecum morphology. Mice fed chow diet, 70S, 50S or 30S showed increased caecum weight compared to mice receiving AIN93G. Caecum weight increased in all modified AIN-based diets (Fig. 1c). Compared to the chow diet, 70S led to increased caecum weight, while 30S and AIN93G led to lower weights (Fig. 1c). Crypt length of mice fed the AIN93G diet was decreased compared to mice fed the chow diet (Fig. 2b). The crypt length of mice fed either the 70S or 30S diet was between the two control diets (chow diet and AIN93G). Mice fed with 50S showed no difference in crypt length to mice fed with AIN93G. We also determined the KI67 proliferation index, to investigate whether the differences in crypt length of the caecum were associated with changes in proliferation. Mice fed the chow diet had a higher proliferation index compared to mice fed 70S, 30S or AIN93G diets (Fig. 2d). Mice of the AIN93G group had a lower proliferation index compared to mice of the 50S diet. In contrast to colon crypt length, caecum crypt length did not correlate with the proliferation index (Fig. 2f).

We further assessed goblet cell counts in caecum crypts. Mice receiving the 70S and the 30S diet had less goblet cells compared to AIN93G-fed mice (Supplementary Fig. S1b). No significant differences were observed between chow diet and any of the other groups.

### Ileum crypt and villi lengths are not affected by different control diets, but goblet cell counts

As we had seen that the different control diets had an impact on colon and caecum morphology, we also wanted to know whether ileum morphology was affected. Yet, no significant differences were observed for ileum crypt length and villus length (Supplementary Fig. S2).

However, ileum goblet cell counts in mice receiving the AIN93G diet were lower compared to chow diet (Supplementary Fig. S1c). The three modified AIN diets were intermediate and differed from both standard control diets. As goblet cell counts were affected by the intervention, we analyzed gene expression of MUC-2, but did not observe any differences (Table 2). In contrast to gene expression of MUC-2, the gene expression of the tight junction proteins JAMA, Occludin and ZO-1 in Ileum was affected by the intervention. For these three tight junction proteins gene expression was higher in mice fed the chow diet compared to AIN93G diet (Table 2). Furthermore, Occludin gene expression in chow-fed mice was also higher compared to the three modified AIN-based diets and JAMA gene expression was higher in chow- compared to 30S-fed mice. No significant differences were observed between AIN93G and the three modified AIN-diet groups.

### Gut microbiota composition and production of SCFA is affected by different control diets

**Figure 3:**
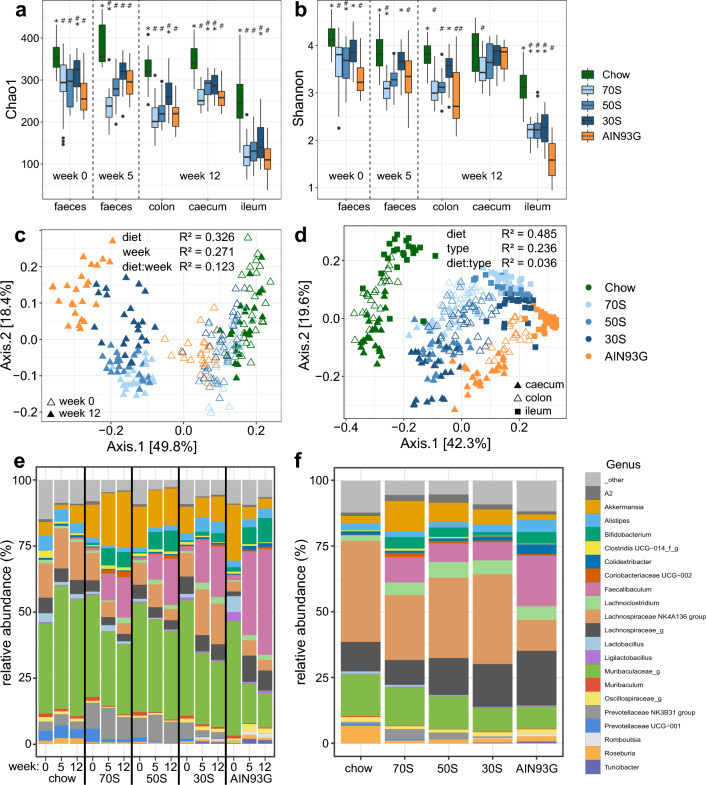
Differences in microbial alpha- and beta-diversity between diet groups (see color legends). (**a**,**b**), Boxplots displaying microbial richness (Chao1 index, **a**) and alpha-diversity (Shannon–Wiener index, **b**) of samples grouped by source material and diet group. * and ^#^ indicate significant difference to AIN93G and Chow, respectively (p < 0.05, Dunnett’s Test). Vertical dashed lines separate samples collected at different time points (indicated as number of weeks). Boxplots display median and quartiles of the data points (n = 24 per group and source material, 23 in faeces of week 0, group “30S”). (**c**,**d**), Principal Coordinates Analysis (PCoA) plots of microbial beta-diversity (measured as Jenson-Shannon divergence) displaying samples of week 0 and week 12 (**c**, feces and colon) and of week 12 across different intestinal compartments (**d**). The fractions of total variance explained by the axes (principal coordinates) are reported as percentages in square brackets. The R^2^ statistic describes the fraction of variance explained by a specific factor in a PERMANOVA analysis comparing group differences in the beta-diversity between samples of different groups. Factors used for grouping were timepoint (‘week’), diet group (‘diet’) and the interaction of both (‘diet:week’) in panel (**c**) and timepoint (‘week’), sample type (= intestinal compartment, ‘type’) and the interaction of both (‘diet:type’) in panel (**d**). Group differences were significant (P < 0.0001, 9999 permutations) for each of the tested variables. (**e**,**f**), bargraphs displaying the average relative abundance of the most abundant genera for each diet group in faeces and colon samples at different timepoints (**e**) and in caecum samples (**f**). Taxa that could not be classified on the genus level were assigned the name of the next highest taxonomic level that could be classified with a ‘_g’ suffix indicating the genus level. 70S: experimental diet with fiber fraction consisting of 70% soluble fiber and 30% cellulose; 50S: experimental diet with fiber fraction consisting of 50% soluble fiber and 50% cellulose; 30S: experimental diet with fiber fraction consisting of 30% soluble fiber and 70% cellulose.

As gut microbiota is directly involved in the utilization of dietary fiber, we also investigated the effects of the different diets on gut microbiota composition and production of SCFA. Chao1 richness index and Shannon diversity index, both measures of alpha-diversity, consistently were highest in mice receiving chow diet (Fig. 3a,b). This effect was already observed in the faecal samples of week 0, only one day after the mice first received the study diets after prior feeding with chow diet. Faecal samples of week 5 and intestinal content that was sampled at the dissections showed more pronounced differences, depending on the compartment. Caecum samples showed the highest diversity but differences in the Shannon index were not significant between the feeding groups. Especially in faecal (week 5) and colon samples, the diversity was reduced from 30S to 50S to 70S group, and closer to the diversity of the AIN93G group (the Shannon index of the 70S was even lower than in AIN93G in faecal samples at week 5).

Differences in the microbial composition (β-diversity) reflected the temporal development and diet dependent differences. Group differences were already visible in faecal samples of week 0 (Fig. 3c) and developed to a larger difference between colon samples of mice receiving chow and AIN93G diets. Colon samples of the 30S, 50S and 70S groups clustered in between the two control groups in the PCoA ordination, with 30S being more similar to the AIN93G samples. The group-wise clustering pattern occurred across the three different intestinal compartments (Fig. 3d), and diet was shown to explain more variance (R^2^ = 0.485) than the intestinal compartment (R^2^ = 0.236) as shown by PERMANOVA analysis (p ≤ 10^–4^). The interaction between intestinal compartment and diet was also significant but explained much less variance (R^2^ = 0.036, p ≤ 10^–4^). A closer look at the microbial composition revealed several diet specific abundance patterns. One group of bacteria including *Faecalibaculum*, *Bifidobacterium* and *Coriobacteriaceae* UCG-002 were only abundant in mice fed one of the purified diets independent from the amount and composition of fiber (Fig. 3e,f, Supplementary Fig. S3 and Supplementary Table S1). Other bacteria were decreased in the purified diets relative to the chow diet. These included *Lactobacillus*, *Prevotellaceae* UCG-001 and *Lachnospiraceae* UCG-001, which all were least abundant in mice fed the AIN93G diet and intermediate in the diets with added pectin and inulin, usually with the highest abundance found in the 70S diet. A third group of bacteria also occurred in higher abundance in mice fed inulin and pectin but in contrast to the previously mentioned group were found in low abundance in mice fed chow diet. This group included *Akkermansia* and unclassified bacteria of the *Prevotellaceae* NK3B31 group. A comparison of samples from feces and colon demonstrates that these differences developed over time but were already visible one day after the start of the study (Fig. 3e, Supplementary Fig. S3b,d).

**Figure 4:**
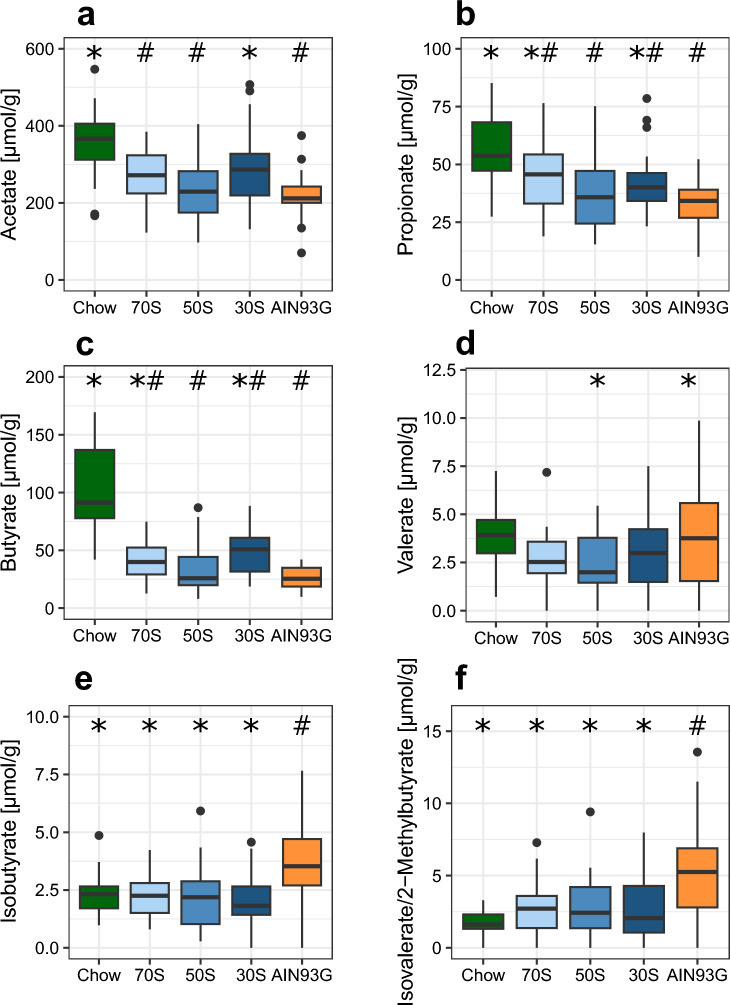
Concentrations of the SCFA acetate (**a**), propionate (**b**), butyrate (**c**) and valerate (**d**) as well as concentrations of the BCFA isobutyrate (**e**) and isovalerate/2-methylbutyrate (**f**) in caecum content of mice fed either chow diet, AIN93G or one of three AIN-based diets with varying ratios of soluble fiber to cellulose. Boxplots display median and quartiles of the data points (n = 24 per group). Values below the limit of quantification are displayed as 0. 70S: experimental diet with fiber fraction consisting of 70% soluble fiber and 30% cellulose; 50S: experimental diet with fiber fraction consisting of 50% soluble fiber and 50% cellulose; 30S: experimental diet with fiber fraction consisting of 30% soluble fiber and 70% cellulose. * and ^#^ indicate significant difference to AIN93G and Chow, respectively (p < 0.05, Dunnett’s Test).

We analyzed SCFA and BCFA concentrations in caecum content as the main site of microbial fermentation in the gut. Concentrations of the three main SCFA acetate, propionate und butyrate were higher in caecum content of mice receiving the chow diet compared to AIN93G diet as well as the three modified AIN-based diets (Fig. 4a–c). No significant differences were observed in valerate concentrations between chow diet and the four other diets (Fig. 4d). Animals receiving the 30S diet had higher concentrations of all three main SCFA compared to AIN93G, whereas no significant differences were observed for animals of the 50S group compared to AIN93G mice. Mice receiving the 70S diet had higher concentrations of propionate and butyrate compared to AIN93G-fed mice. Furthermore, mice receiving the 70S and the 50S diets had lower concentrations of valerate in caecum content compared to AIN93G-fed mice.

In contrast to SCFA concentrations, concentrations of the BCFA isobutyrate and isovalerate / 2-methylbutyrate were higher in AIN93G-fed mice, compared to the four other groups (Fig. 4e,f). No significant differences were observed between the chow diet and the three modified AIN-based diets.

In addition, we analyzed SCFA and BCFA concentrations in faeces at week 10. The concentrations of acetate, propionate and butyrate were much lower than in the caecum (see also Supplementary Fig. S4), but the diet-specific pattern was similar. Acetate, propionate, and butyrate concentrations were also higher in the chow diet group compared to the AIN93G group, while the 70S, 50S and 30S diets had intermediate concentrations. Valerate and BCFA could not be detected in most faecal samples.

**Figure 5:**
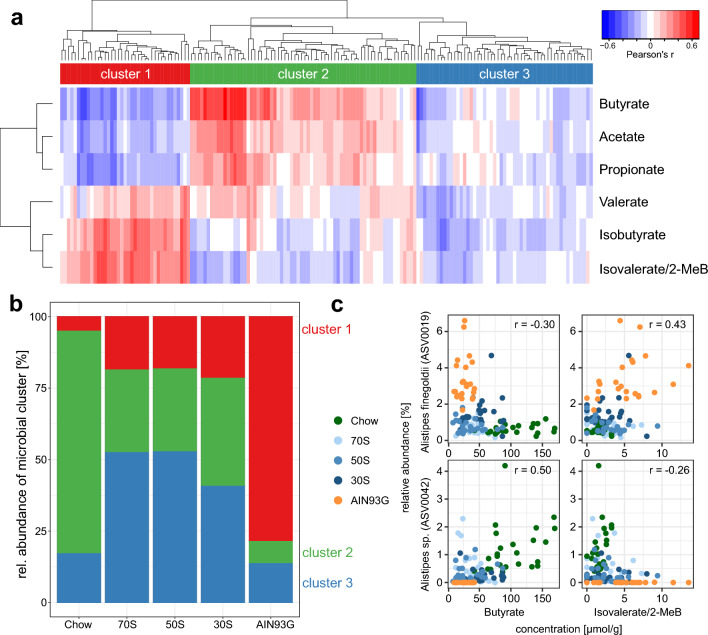
Correlations between microbiota and SCFA/BCFA profiles in the caecum. (**a**) Heatmap of correlation coefficients (Pearson’s r) between relative abundances of microbial taxa and concentrations of SCFA and BCFA in the caecum. Taxa were grouped by hierarchical clustering into three clusters as indicated by the colorbar above the heatmap. (**b**) Average relative abundances of the three clusters of taxa that were determined from the correlation with SCFA/BCFA (clusters correspond to panel **a**). (**c**) Comparisons of concentrations of the SCFA butyrate and the BCFA isovalerate/2-methylbutyrate with relative abundances of two selected ASVs of genus *Alistipes* as an example of microbe-metabolite association. ASV0019 is part of the red cluster and ASV0042 part of the green cluster. Correlation coefficients are indicated for each graph (r).

An analysis of the correlations between abundances of individual microbial taxa and concentrations of SCFA and BCFA in the caecum revealed three clusters of microbial taxa that were positively correlated either with SCFA or BCFA concentrations (Fig. 5a, green cluster or red cluster, respectively, see also Supplementary Table S2) or not showing pronounced correlations (blue cluster). The cecal abundances of bacteria of these clusters were different between mice receiving different diets (Fig. 5b). Bacteria positively correlated with SCFAs were found most abundantly in mice receiving chow diet and those correlated with BCFA were most abundant in the AIN93G group. As an example of the associations between microbial taxa and metabolite concentrations, Fig. 5c displays the correlation between two sequence variants of *Alistipes* with butyrate and isovalerate/2-methylbutyrate.

SCFA are known as energy substrates for colonocytes and therefore can impact cell growth^10^. Because of this, we investigated whether caecum SCFA concentrations were associated with crypt length and KI67 proliferation index in caecum and colon. Weak correlations between the caecum concentrations of acetate, propionate and butyrate and the colon crypt length were found. However, a correlation between colon KI67 proliferation index and SCFA concentrations that significantly differed from zero was not observed (Supplementary Fig. S5a,b). In caecum moderate correlations were observed for SCFA concentrations and crypt length (Supplementary Fig. S5c). Furthermore, butyrate and propionate correlated moderately with the cecal KI67 proliferation index (Supplementary Fig. S5d).

## Discussion

Even though the choice of (control) diets in animal studies has a huge impact on study outcomes, a careful choice of diet is often neglected and information on diet in publications are often lacking^1^. Purified diets seem to be a better alternative for more standardized studies, as they have open formulas and can be manufactured without batch-to-batch variation. However, most purified diets contain much less dietary fiber compared to grain-based chow diets and typically soluble fiber is lacking completely^4,8^. It is known that differences in gut morphology between animals fed chow diets compared to purified diets occur and it has been suggested that the dietary fiber fraction of purified diets might not support optimal gut health^8^. Thus, diets that ensure high scientific quality, meaning reproducibility and reportability as well as good physiological properties are lacking. Given the importance of diet for both aspects, this problem should be addressed. Therefore, the aim of the present study was, to modify the purified diet AIN93G using dietary fiber, in order to achieve gut development as seen with a grain-based chow diet. We increased dietary fiber content from 5 to 15% and used different ratios of soluble to insoluble fiber (70:30, 50:50 and 30:70). As we did not want to impair reproducibility, we decided to use two well standardized fiber products. Furthermore, with pectin and inulin we aimed to use widespread soluble fibers.

Despite some differences in the energy uptake depending on the diet, all mice gained weight at the same rates indicating normal general growth and development. Our main interest were morphological changes in the gut, particularly in the colon. The results show clear morphological differences between the two diets commonly used as control diets, namely chow diet and AIN93G. Colon length and colon crypt length, as well as caecum weight and caecum crypt length were decreased in mice receiving the AIN93G diet compared to the chow diet, indicating gut atrophy in mice fed purified diets. Our results are in line with a previous study, which also showed decreased colon and caecum growth in mice fed purified diets compared to chow diet^7^. Supplementation of AIN93G with (soluble) fiber mitigated the differences, which indicates that the addition of soluble fiber to purified diets is necessary for regular gut development. The underlying mechanism might involve an increased proliferation, as our results further show, that the decreased tissue atrophy was associated with an increased proliferation. In contrast to colon and caecum, no significant differences in ileal villus and crypt length were observed.

Since microbial fermentation of dietary fiber primarily takes place in the large intestine and caecum, it could be that fermentation products mitigate the observed effects, which are lacking in ileum and thus do not influence ileum morphology.

Tight junctions are important for the regulation of gut barrier function, as they seal the paracellular space of the epithelial cell layer and regulate paracellular transport^12^. It has been shown that SCFA promote epithelial barrier integrity by increasing tight junction protein expression^13^. Therefore the gene expression of several tight junction proteins were also analyzed. Gene expression of tight junction proteins in mice fed chow diet was higher compared to AIN93G, especially in the ileum. This might indicate a better gut barrier function. However, as the observed differences were very small, it is questionable if they actually have any biological relevance.

The availability of substrates for microbial fermentation shapes the gut microbiota composition, with dietary fiber playing a major role in this process. In turn the gut microbiota has a huge impact on host physiology^10^. Because of this, we were interested in the gut microbiota composition and its fermentation products. Our results demonstrate that microbiota from mice receiving chow diet and AIN93G diet are clearly different. The three fiber-supplemented AIN-based diets seem to shift the composition towards the state observed for the chow diet. Our analyses further show that diet was the most important factor causing variation of the microbiome, which again stresses the importance of control diet choice, especially for studies focusing on gut microbiota. Higher microbial α-diversity is often considered a marker of a healthy intestinal microbiota^14,15^. Our results show, that already after one day on AIN93G diet, the α-diversity is reduced compared to chow diet and consequently the microbiome composition differed. The animals were randomly assigned to the diet groups directly upon arrival and first fecal samples were collected only one day later to reduce stress for the animals. Although the situation before the start of the study can only be assumed, the α- and β-diversities for week 0 are consistent with the interpretation that microbiomes were similar before and quickly adapted after the start of the diets. Dietary groups clustered visibly and AIN93G fed mice deviated most from the chow group in week 0. Somewhat surprisingly, the Shannon index did not show significant differences in caecum content between mice fed chow diet or AIN93G. Apparently, high diversity of the cecal microbial community does not require soluble fiber but the differences in the microbial and metabolic profiles indicate a substantial difference in microbial activity in response to the different diets. Interestingly, the biggest differences in α-diversity were observed in the ileum. As fiber fermentation takes place primarily in the caecum and colon, other factors than fiber fermentation play a role in the modulation of ileal microbiota composition. It is well known that dietary fiber can bind bile acids and lead to an increased bile acid excretion^16^. Further, it is known that bile acids can influence microbiota composition^17^. Specifically, inulin has been shown to promote microbiota-derived bile-acids and alter the composition of the microbiota^18^. Thus, the increased concentrations of fiber, especially inulin in the gut lumen could have led to changes in concentrations of bile acids, which in turn influenced gut microbiota composition.

Common products of microbial fermentation are SCFA and BCFA, which can have an impact on host physiology^10,19,20^. As expected, the concentrations of the three main SCFA acetate, propionate and butyrate were increased in mice receiving the chow diet compared to AIN93G. Especially butyrate concentrations were higher. This is of interest, because butyrate is well known to promote gut health as reviewed elsewhere^21^. Whereas SCFA are products of dietary fiber fermentation, BCFA are known products of protein fermentation^22^. Our results show, that BCFA concentrations were increased in mice receiving the AIN93G diet, which could indicate an increase in protein fermentation in the absence of fermentable dietary fiber. As reviewed elsewhere^22^ fermentation of proteins due to lack of fiber leads to reduced concentrations of SCFA and increased concentrations of BCFA, as well as further metabolites such as ammonia or sulfides, which have been associated with the development of colon cancer and chronic noncommunicable diseases. Therefore, the lack of fermentable fiber in purified diets might have severe health impacts. In this study, the addition of inulin and pectin to the diets led to increased concentrations of SCFA and reduced concentrations of BCFA compared to AIN93G and changed microbiota composition towards bacteria belonging to the intermediate fermentation cluster (Fig. 5b). The example of two variants of the genus *Alistipes* (Fig. 5c) shows associations with SCFA and BCFA profiles that might indicate their active contribution. This demonstrates the limitations of the 16S-rRNA based taxonomic profiling on the one hand and of our knowledge of intestinal microbes on the other hand, as both variants likely represent uncharacterized species. Further, it underlines the importance of using complementary methods to determine microbial activity, e.g., metabolic profiling.

The diet-specific patterns of microbial abundances could partially be explained by the differences between the complex chow diet and the less complex purified diets. Bacteria like *Lactobacillus* and *Prevotellaceae* UCG-001 only grow well in diets containing a mixture of soluble fibers and decrease in the purified diets that contain only inulin and pectin or no soluble fiber at all in our experiment. This corresponds well with our previous study comparing various fractions of wheat grain. *Lactobacillus* and *Prevotellaceae* abundance increased in a diet supplemented with wheat bran or aleurone which are rich in complex fibers, compared to the starch-dominated flour fraction^23^. In contrast, bacteria like *Faecalibaculum* and *Bifidobacterium* apparently benefit from the purified diets, which might be due to the lack of competition from other bacteria or the promotion of their growth due to specific ingredients of the purified diets. Bifidobacterium was shown to increase upon high fructose diets or maltodextrin^24,25^. All AIN diets are richer in simple sugar and maltodextrin than the chow diet, what might explain the increase in Bifidobacterium. The third group mentioned above including *Akkermansia* and the *Prevotellaceae* NK3B31 group seems to specifically depend on the presence of inulin and pectin, since their abundances increased with the amount of these two soluble fibers but remained low in the chow diet.It was previously reported that *Akkermansia* abundance increased in response to inulin rich diets^26,27^. This genus is known to grow by degradation of mucins but not dietary fiber. The number of goblet cells determined in this study did not correlate with the abundance of *Akkermansia*, so there is no evidence for an increased mucus production that could explain the expansion of this species. The response to the fiber-rich diets must involve more complex interactions, probably competition for ecological niches as was hypothesized previously^28,29^, or maybe cross-feeding in connection with primary degraders such as *Prevotellaceae*. In addition, caecum weight is increased and for the 70S group even higher than in mice receiving chow diet (Fig. 1c), while proliferation in this group is reduced (Fig. 2d). It is currently unclear if this implies potentially negative effects of a diet (too) high in soluble fiber with low complexity (i.e., few kinds of different fibers) and thus requires further investigation.

As SCFA are energy substrates for colonocytes, they can impact their growth. In our study both, crypt length and proliferation indices, in colon and caecum correlated positively with concentrations of butyrate. Therefore, we hypothesize that inulin and pectin as soluble fibers were fermented to SCFA, especially butyrate, which led to increased proliferation and healthy development of gut tissue. This would further explain, why we did not observe any changes in ileal villus and crypt length.

Our results show clear differences between a grain-based chow diet and the standard purified diet AIN93G, thus highlighting that the interpretation of study results depends on the control diet used. We conclude that scientists need to pay more attention to this aspect when designing studies. To our knowledge this is the first study that tried to improve a purified diet in order to mimic physiological gut development as seen with a chow diet and thus combining the advantages of both diet types. The addition of the soluble fibers inulin and pectin successfully mitigated some of the differences. However, none of the three AIN-based diets overcame the differences completely and a clear dose–response relationship was not observed. Thus, there is still potential for improvement. One aspect that should be investigated in future studies is the use of a more complex fiber mixture. Considering that the use of inulin and pectin promoted the growth of some bacterial species indicating an overrepresentation of a very specific ecological niche, such a simple supplementation can mitigate some of the differences between chow and AIN93G diets but can also lead to other unwanted side effects.

In summary, our results indicate that the lack of soluble dietary fiber in standard purified diets such as the AIN93G leads to morphological changes in the gut and a less diverse gut microbiota in combination with a reduced SCFA production. Moreover, the low SCFA concentrations seem to support gut tissue atrophy. Thus, our study stresses the importance of the addition of soluble fiber to purified diets. However, an ideal fiber mixture still needs to be developed.

## Methods

### Study design

Four-week-old male C57BL/6J mice (wildtype; WT) (average initial weight, 10.12 g ± 1.34 g) were purchased from Charles River (Sulzfeld, Germany) and were kept under specific pathogen-free conditions (SPF- FELASA health monitoring recommendations) in a 12-h light–dark cycle and a temperature-controlled (22 ± 1 °C) environment. Cages were changed weekly. All experimental protocols were approved by Regierungspräsidium committee (regional council Karlsruhe #35-9185.81/G-223/19) and carried out in the Central Animal Facility of the Max Rubner-Institut Karlsruhe in accordance with the German animal protection law. All methods are reported in accordance with Animal Research: reporting in vivo experiments (ARRIVE) guidelines^30^.

Immediately after arrival, 120 animals were randomly assigned to the five different diet groups (24 mice/group, 8 cages/group with 3 mice/cage). No animal had to be excluded throughout the study. Details for randomization and biometric planning of the study are given below. Mice received the diets and water *ad libitum* during the 12-week study period. The five diets consisted of either an experimental control diet (standard chow diet from Ssniff (V1534-000) [Chow] or AIN93G diet [AIN93G]) or a modified AIN93G diet with increased dietary fiber content (15% wt:wt) with different ratios of soluble fibers to cellulose (70% soluble fiber:30% cellulose [70S], 50% soluble fiber:50% cellulose [50S] or 30% soluble fiber:70% cellulose [30S]). In order to have a comparable total fiber content in the modified AIN93G diets compared to the chow diet, we increased the fiber content in all modified AIN93G diets to 15%. This is approximately the same as the total fiber content of commercially available chow diets.

Pectin and inulin are well known for their positive effects on gut health. The soluble fiber fraction consisted of 50% apple pectin (“Classic AU 202” from Herbstreith und Fox) and 50% inulin (Orafti®Synergy1 from Beneo). Orafti® Synergy1 is a dedicated configuration of long- and short-chain inulin from chicory roots, which can be fermented at different speed in the gut^31^, therefore potentially nourishing the microbiota at different places along the gut. All diets were produced by Ssniff.

Insoluble, soluble, and total dietary fiber were analyzed by the official AOAC method 2011.25, using the optimized protocol of McCleary et al.^32^. Briefly, nonresistant starch and proteins were digested enzymatically. IDF (insoluble dietary fiber) and the alcohol-insoluble fraction (SDFP; soluble dietary fiber, precipitated) were analyzed gravimetrically, and corrected for protein content (assessed via nitrogen determination according to Kjeldahl). The water-soluble fraction (SDFS; soluble dietary fiber, in solution) was purified by ion exchange columns (Amberlite/Ambersep, Megazyme) and analyzed by Gel-HPLC (TSKgel G2500PWXl, 7.8 × 300 mm, Tosoh Bioscience) on an Agilent 1100 HPLC–DAD/RID. Total fiber was calculated as sum of all fractions. The exact composition of the experimental diets is given in Table 3 and Supplementary Table S3.

**Table 3:** Fiber content (AOAC 2011.25) and composition of experimental diets [% wt:wt] according to the manufacturer’s data sheets.

	Chow	70S	50S	30S	AIN93G
Measured fiber content					
TDF	30.6	16.2	16.6	16.2	9.2
IDF	21.9	5.0	7.8	9.9	5.8
SDFP	5.0	6.6	5.4	4.3	2.5
SDFS	3.6	4.6	3.4	2.0	0.9
Dry weight %	89.5	89.4	89.4	89.6	88.5
Nutrient content according to data sheets					
Crude fiber	5.0				
NDF	16.9				
ADF	7.1				
Cellulose		4.5	7.5	10.5	5.0
Pectin		5.25	3.75	2.25	-
Synergy 1		5.25	3.75	2.25	-
Crude protein	19.0	17.6	17.6	17.6	17.6
Crude fat	3.3	7.1	7.1	7.1	7.1
Crude ash	6.4	3.2	3.2	3.2	3.2
Starch	35.9	33.4	33.4	33.4	38.2
Dextrin		10.6	10.6	10.6	13.1
Sugar	5.4	9.1	9.0	8.9	11.2
Energy [MJ/kg]	13.5	14.7	14.6	14.6	16.2
Fat [MJ%]	9	18	18	18	17
Protein [MJ%]	24	20	20	20	18
Carbohydrates [MJ%]	67	62	62	62	65

*TDF* total dietary fiber, *IDF* insoluble dietary fiber, *SDFP* soluble dietary fiber, precipitated, *SDFS* soluble dietary fiber, in solution, *ADF* acid detergent fiber, *NDF* neutral detergent fiber.

70S: experimental diet with fiber fraction consisting of 70% soluble fiber and 30% cellulose.

50S: experimental diet with fiber fraction consisting of 50% soluble fiber and 50% cellulose.

30S: experimental diet with fiber fraction consisting of 30% soluble fiber and 70% cellulose.

During the intervention, body weight and food intake were assessed four times per week. Feces was collected during week 0, week 5, and week 10. After 12 weeks on the respective diets, animals were anesthetized with ketamine (100 mg/kg) and xylazine (10 mg/kg) and terminated by cardiac puncture. Energy intake of each diet was calculated as follows: ([total administered food per cage] − [total consumed food per cage])/([number of animals in cage] * [duration of intervention]). Complete colon tissue was collected, emptied and colon length measured on an ice-cold plate. Caecum was weighted and caecum content collected and snap frozen in liquid nitrogen. All tissues used for gene expression analysis were cut in small pieces, immersed in RNAlater (Thermo Fisher Scientific) and snap frozen in liquid nitrogen. All tissues for histological analysis were fixed in formalin.

### Histology/immunohistochemistry

Formalin-fixed paraffin embedded tissues were sectioned (3 µm), placed on glass slides, deparaffinized and stained with either Hematoxylin and Eosin (HE) or Alcian Blue and Nuclear Fast Red (AB). To evaluate crypt cell proliferation, we assessed KI67 protein by immunohistochemistry. Pictures of stains were obtained on a Zeiss AxioScope 5 microscope equipped with a Zeiss AxioCam 503 camera and ZEN blue software version 3.1 in × 400 magnification. For all measurements Image J software version 1.51w was used. For further details see Supplementary Methods S1.

### Gene expression

Colon and ileum tissues were stored in 300 µL RNAlater at − 80 °C. For gene expression analysis 30 mg of each tissue were homogenized. After centrifugation supernatants were used for RNA isolation with RNeasy Mini Kit (Qiagen, #74106), according to the manufacturer´s protocol followed by Dnase digestion. cDNA synthesis and qPCR amplification were performed using the GoTaq® 2-Step RT-qPCR system kit (Promega #A6010), according to the manufacturer´s protocols. The cDNA products were subjected to SYBR-Green-based real-time amplification using the LightCycler®480 (Roche). Primer sequences and specific conditions are listed in Supplementary Table S4. Different reference genes for normalization were used for each tissue. The expression level of different targets, relative to reference genes and relative to the study calibrator (composed of 25 mice; 5 mice from each group), was determined by using the E-Method (Roche)^33^. For the assessment of the intestinal barrier, we analysed the gene expression of tight junction proteins, junctional adhesion molecule F11 (JAMA), Occludin and Zona occludens-1 (ZO-1) as well as the Mucin expression gene (MUC-2). A detailed description of the protocol can be found in the Supplementary Methods S1.

### Intestinal microbiome analysis

Microbiome analysis was performed on feces samples collected one day after the start of the dietary intervention (= week 0) and after 36 days (= week 5) and on samples collected during dissection (= week 12) from the ileum, caecum, and colon. DNA was isolated from each sample using a bead-beating method based on the NucleoSpin DNA Stool kit (Macherey–Nagel). The variable region V4 of the 16S rRNA gene was amplified using dual indexing primers as described by Kozich et al. Microbiome data analysis was performed in R using *dada2*^35^ to determine amplified sequence variants (ASVs), the Silva database v138.1^36^ for taxonomic classification and *phyloseq*^37^ for downstream analysis and visualization. Detailed descriptions of the microbiome analysis from sample collection to bioinformatics analysis can be found in the Supplementary Methods S1.

Raw sequence data have been deposited in the European Nucleotide Archive ENA^38^ under the accession number PRJEB54038.

### GC-analysis of SCFA and BCFA in caecum content and feces

SCFA and BCFA were quantified in caecum and feces samples by GC-FID without derivatization, using a slightly modified version of the method described in^23^. Briefly, about 100 mg of caecum or feces were freeze-dried (Alpha 1–2, Martin Christ) and extracted with aqueous HCl in presence of 4-ethylbutyric acid as internal standard. Extracted SCFA/BCFA were quantified by GC-FID on a 6890 GC (Agilent) equipped with a split/splitless injector (split 1:10). Compounds were separated on a water-tolerant WAX column (ZB-WAXplus, 30 m × 0.25 mm × 0.25 µm; Phenomenex) using hydrogen as carrier gas. Further method details can be found in the Supplementary Methods S1.

### Randomization, biometrical planning and statistical analysis

Data were statistically analyzed by the software R, version 4.1.2 (2021-11-01), if not differently stated.

Before starting the study, sample size was calculated with the primary outcome being the colon length based on a Dunnett’s type test with equivalence hypothesis with one control and three treatment groups. With a significance level of 5%, a power of 80% at least 24 animals per group were needed, taking into account, a comparable husbandry of 3 animals per cage. Comparable means that the “with respect to the diet groups nested cage effect” was modelled as a random effect and was assumed to be independent of the diet groups. The calculation was based on the following assumptions: (i) a comparable effect as seen in Chassaing and co-workers^7^, in which mice (male wild type C57BL/J6 mice) were fed a standard laboratory chow diet for 12 weeks and showed an arithmetic mean of 8.8 mm with a standard deviation of 0.6 mm for the colon length; (ii) using a equivalence threshold based on a deviation of 0.6 mm; (iii) considering expected values for the colon lengths of 7.6 mm, 8.2 mm, 8.74 mm (represent effect sizes 2, 1 and 0.1) for the three high-fiber diets (30S, 50S, 70S) to be plausible.

Randomization was conducted to assign mice to groups and for the order of dissection on each day. Therefor the R-base-function *sample* was mainly repetitive used.

For statistical analysis, descriptive summary statistics were calculated and data were visualized via boxplots. Most of the parameters were continuous variables. Normal distribution of the parameters was assumed, and graphically checked by Q–Q-plots. Experimenter and statisticians discussed measurements which were possibly not valid. These were excluded from data processing. As descriptive and explorative analysis, correlations between caecum concentrations of acetate, propionate, and butyrate and the colon crypt length, the caecum crypt length the colon KI67 proliferation index and the colon KI67 proliferation index were calculated. Calculation of correlations were made with the software sigmaPlot. Specifically, the sigmaPlot function “pearson product moment correlation” was applied, as parametric test (under assumption of normal distribution). Then, linear mixed models were fitted (R function *lmer*, package *Ime4*)^39^ with treatment groups as fixed effects and cages as random effects. As explorative analysis, we conducted Dunnett's tests for comparisons of the treatment groups versus a control group (the script and a demonstrative example are available under 10.5281/zenodo.8070254). Specifically, two approaches were employed to assess the significance of pairwise comparisons between the treatment and the control groups: the control diet was the AIN93G diet or the chow diet. Simultaneous confidence intervals for the estimated effects were calculated. Significance level was set at p < 0.05. Differences in beta diversity were tested using Permutational Analysis of Variance (PERMANOVA) with the function *adonis2* of the *vegan* package^40^. Week 0 feces samples were compared with week 12 colon samples including the week and diet as factors and samples across different parts of the intestine were compared using the sample type (intestinal compartment) and diet as factors. The cage ID was used in PERMANOVA analysis as a constraint for permutations (using the *strata* argument) to account for cage specific variation. Differences in the abundance of individual bacterial taxa between diets were tested using centered log transformations and a generalized linear model using diet as with the *ALDEx2* package^41^.

All results in this article are presented as arithmetic mean and standard deviation, if not stated otherwise.

## Supplementary Information

Supplementary Information 1.

Supplementary Tables.

## Data Availability

Data of the measurements mentioned in this study have been deposited in OpenAgrar (10.25826/Data20230821-143408-0). Sequence data of the microbiome amplicon sequencing are available from the European Nucleotide Archive (ENA, https://www.ebi.ac.uk/ena) under Accession No. PRJEB54038. A script to perform the Dunnett's test has been deposited in Zenodo with simulated example data (10.5281/zenodo.8070254).
