# Correction: Addition of soluble fiber to standard purified diets is important for gut morphology in mice

**DOI:** 10.1038/s41598-026-59195-2

**Published:** 2026-06-22

**Authors:** Marietta von Süßkind-Schwendi, Andreas Dötsch, Vivien Haberland, Paola Ferrario, Ralf Krüger, Sandrine Louis, Maik Döring, Daniela Graf

**Affiliations:** 1 Department of Physiology and Biochemistry of Nutrition, Max Rubner-Institut (MRI)-Federal Research Institute of Nutrition and Food, Haid-Und-Neu-Straße 9, 76131 Karlsruhe, Germany; 2 National Reference Centre for Authentic Food, Max Rubner-Institut (MRI)-Federal Research Institute of Nutrition and Food, E.-C.-Baumann-Straße 20, 95326 Kulmbach, Germany

Correction to: *Scientific Reports* 10.1038/s41598-023-46331-5, published online 07 November 2023

The original version of this Article contains errors in Supplementary Figures 3 and 4 located in Supplementary Information 1.

Supplementary Figure S3 is rendered poorly and therefore the text is difficult to read.

In Supplementary Figure S4, during calculation of short chain fatty acid concentrations in fecal samples, the values were normalized using water content instead of dry weight. Correcting the values revealed minor changes and some additional comparisons ultimately showing statistically significant differences.

The Supplementary Information is now linked to this correction notice.

## Supplementary Information

Supplementary Information 1.

