# Supplementary material for: Correction: Addition of soluble fiber to standard purified diets is important for gut morphology in mice
Source: Sci Rep. 2026 Jun 22;16:19335. doi: 10.1038/s41598-026-59195-2 (PMC13287475; doi:10.1038/s41598-026-59195-2)
Supplement: Supplementary file 1 — Supplementary Information 1. [file 41598_2026_59195_MOESM1_ESM.pdf]

Supplementary Information

Supplementary Results

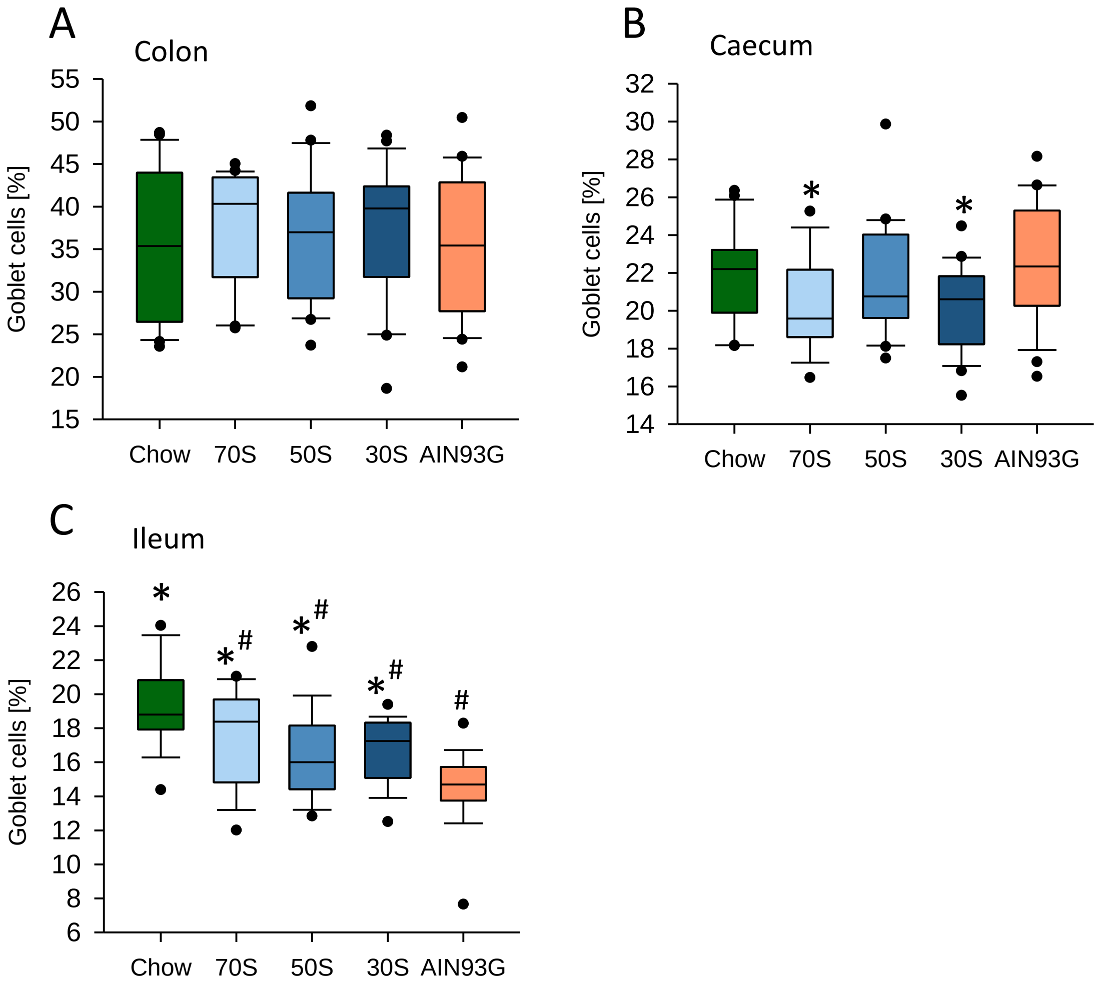

Supplementary Figure S1: Percentage of goblet cell in colon (A), caecum (B) and ileum (C) in cells lining the crypts.

Mice were fed either chow diet, AIN93G or one of three AIN-based diets with varying ratios of soluble fiber to cellulose for 12 weeks.

70S: experimental diet with fiber fraction consisting of 70% soluble fiber and 30% cellulose; 50S: experimental diet with fiber fraction consisting of 50% soluble fiber and 50% cellulose; 30S: experimental diet with fiber fraction consisting of 30% soluble fiber and 70% cellulose.

* and # indicate significant difference to AIN93G and Chow, respectively ($p < 0.05$, Dunnett's Test).

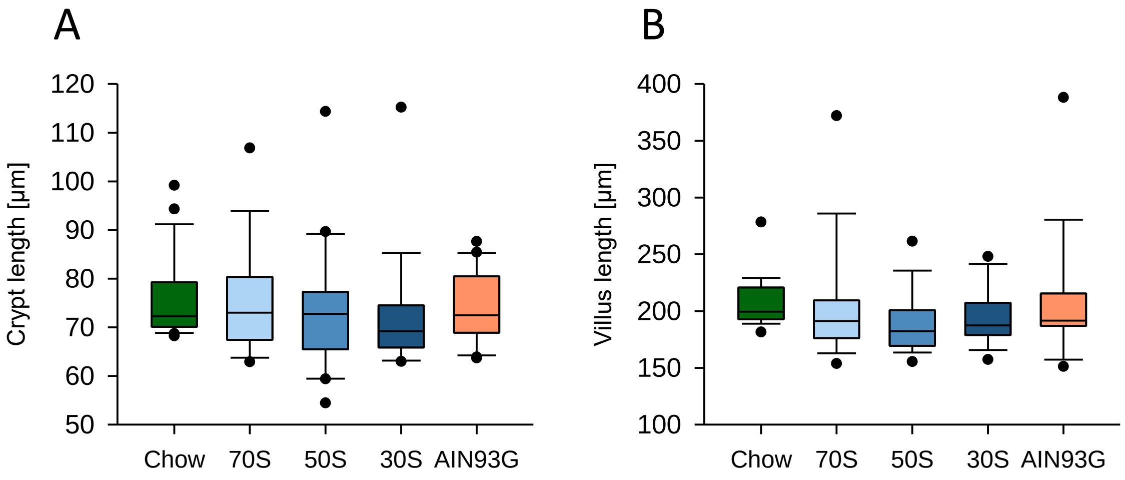

Supplementary Figure S2: Ileum crypt length (A) and villus length (B) of mice fed either chow diet, AIN93G or one of three AIN-based diets with varying ratios of soluble fiber to cellulose.

70S: experimental diet with fiber fraction consisting of 70% soluble fiber and 30% cellulose; 50S: experimental diet with fiber fraction consisting of 50% soluble fiber and 50% cellulose; 30S: experimental diet with fiber fraction consisting of 30% soluble fiber and 70% cellulose.

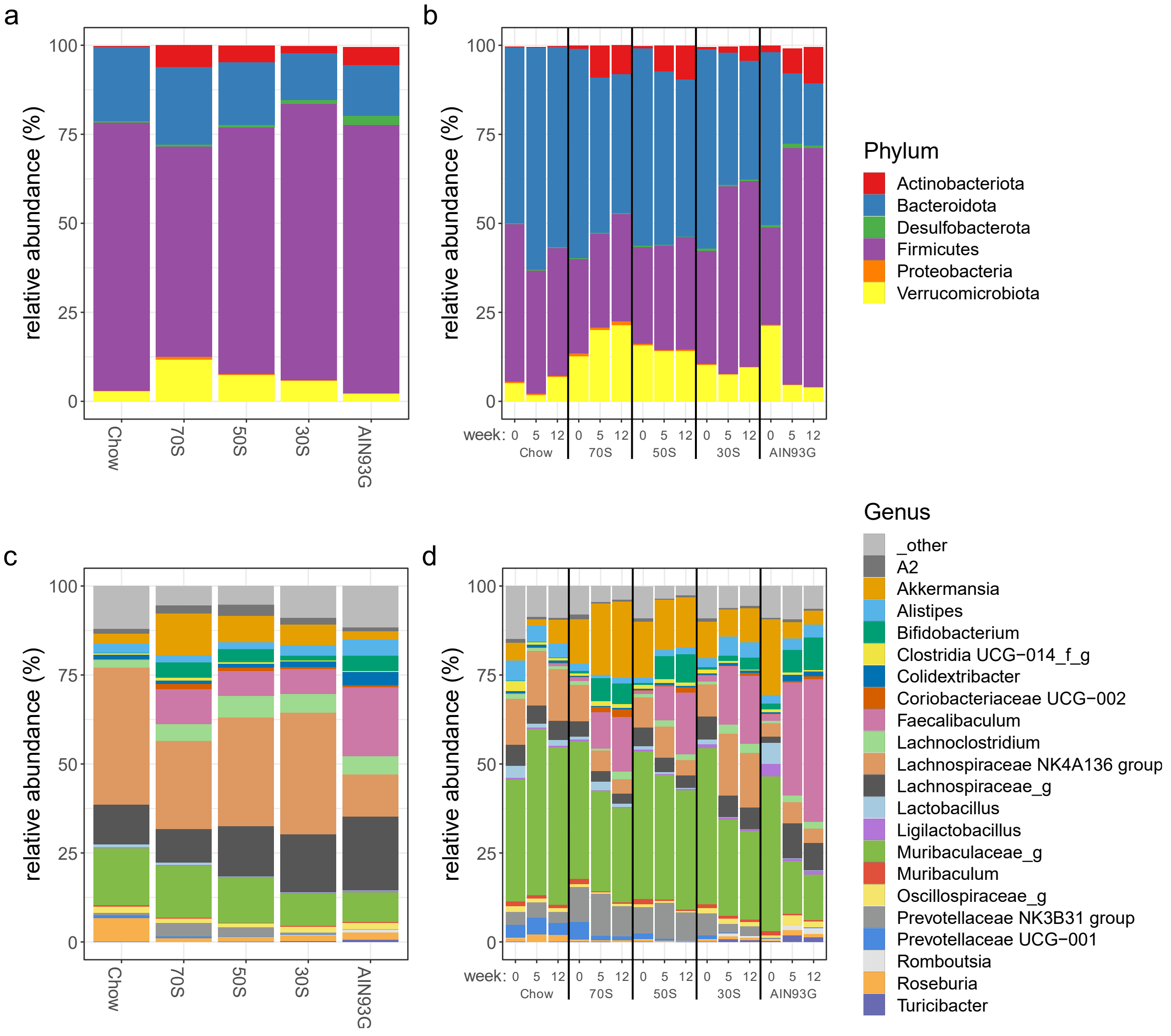

Supplementary Figure S3: Average microbiome composition in caecum samples (A, C) and faeces/colon samples at different timepoints (B, D), and different taxonomic level (Phyla A, B; most abundant genera C, D)

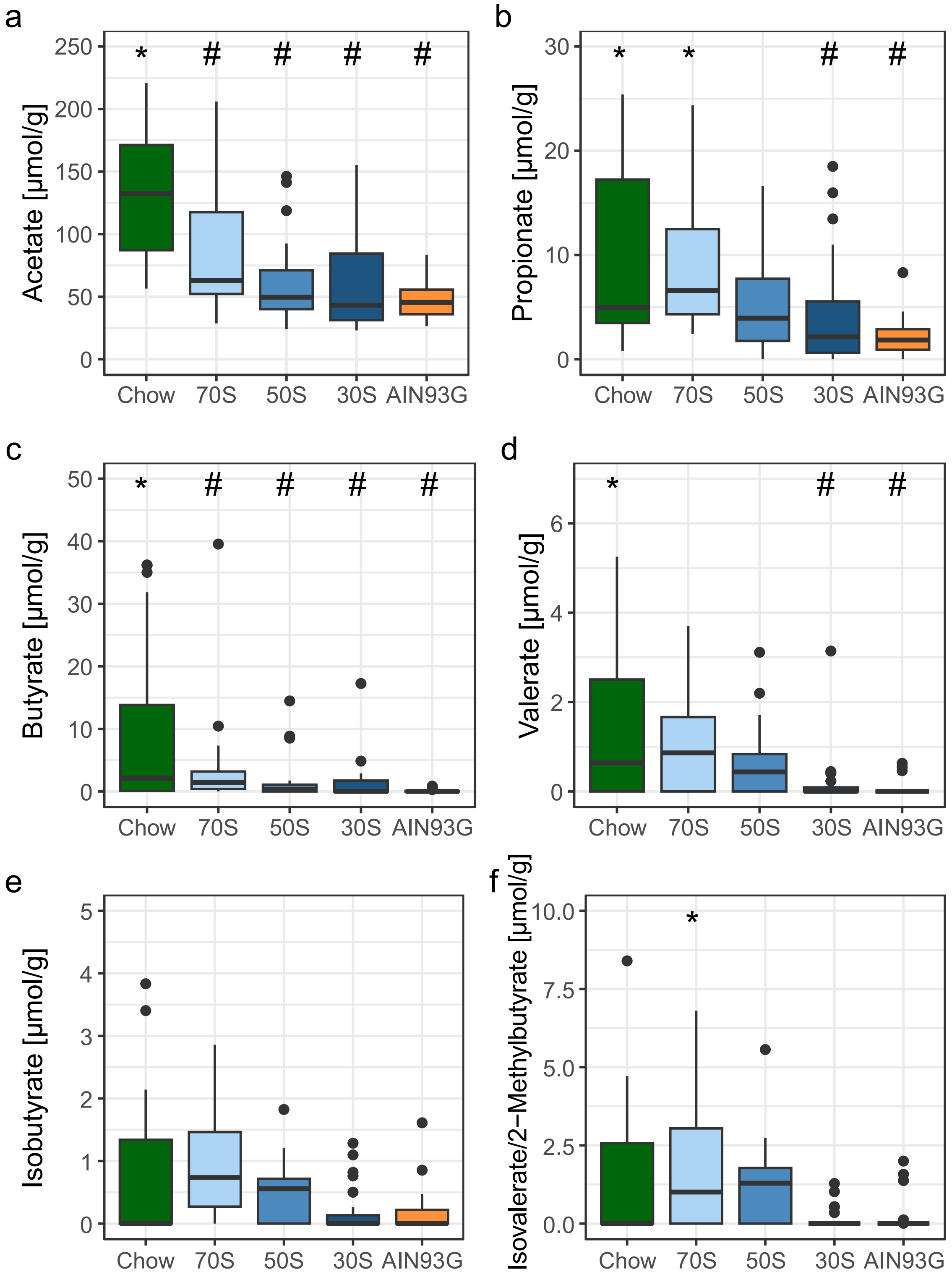

Supplementary Figure S4: Concentrations of the short chain fatty acids (SCFA) in faeces of mice fed either chow diet, AIN93G or one of three AIN-based diets with varying ratios of soluble fiber to cellulose.

70S: experimental diet with fiber fraction consisting of 70% soluble fiber and 30% cellulose; 50S: experimental diet with fiber fraction consisting of 50% soluble fiber and 50% cellulose; 30S: experimental diet with fiber fraction consisting of 30% soluble fiber and 70% cellulose.

* and # indicate significant difference to AIN93G and Chow, respectively ($p < 0.05$, Dunnett's Test).

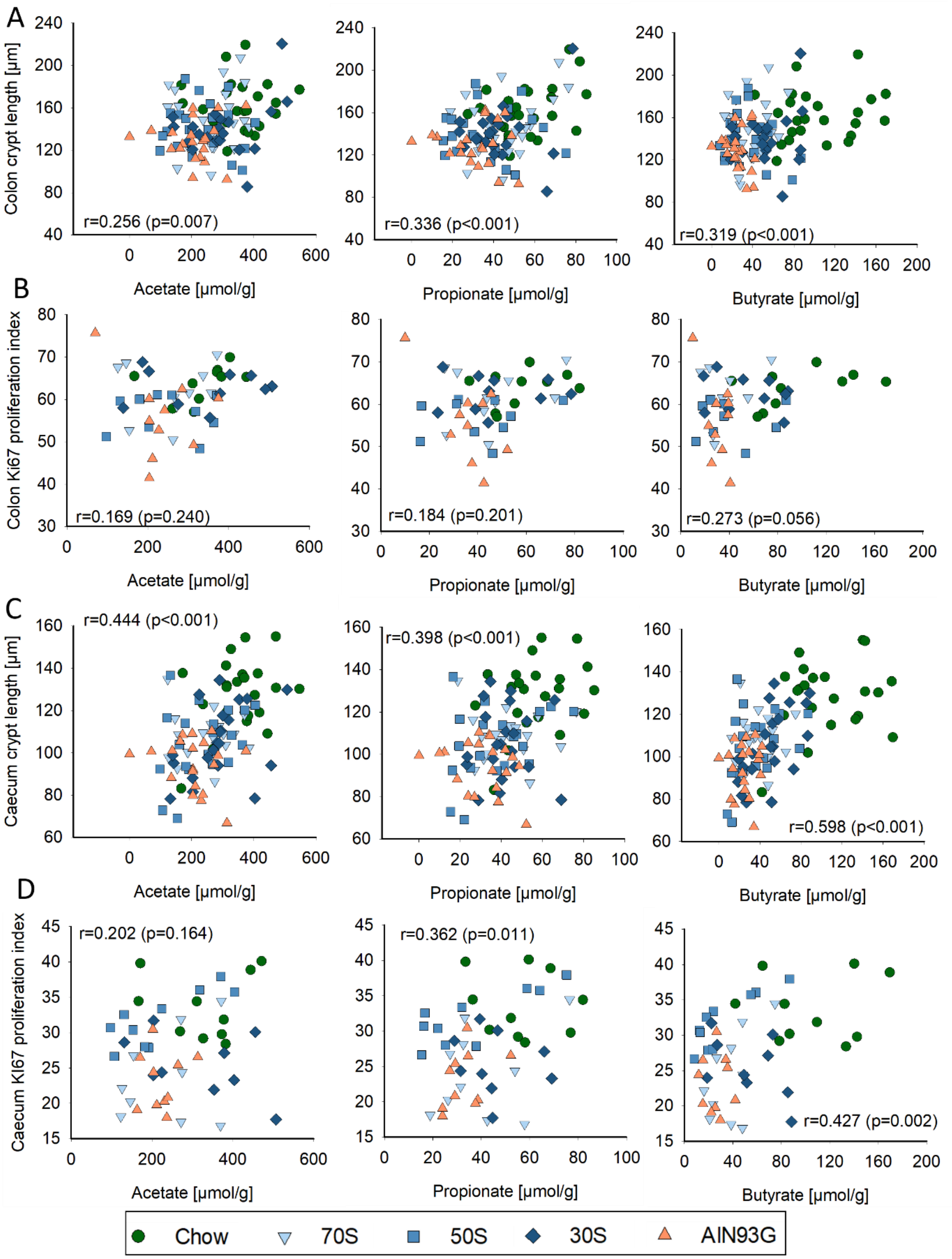

Supplementary Figure S5: Scatter blots of colon crypt length (A) , colon proliferation index (B), caecum crypt length (C), caecum proliferation index (D) and the caecal concentrations of the three main SCFA acetate, propionate and butyrate.

70S: experimental diet with fiber fraction consisting of 70% soluble fiber and 30% cellulose; 50S: experimental diet with fiber fraction consisting of 50% soluble fiber and 50% cellulose; 30S: experimental diet with fiber fraction consisting of 30% soluble fiber and 70% cellulose.

Supplementary Methods:

Histology

Crypt length was measured in colon, ileum and caecum from crypt base to gut lumen using the segmented line tool in Image J (Supplementary Figure 5). For measurements in colon and ileum two cross-sections from within 1 cm of gut tissue were used. In colon 20 measurements were made, in ileum 10 - 20 and in cecum 8 - 20. For villus length in ileum cross-sections 8 - 20 villi were measured accordingly. In AB-stained sections of colon, ileum and caecum goblet cells and epithelial cells lining the crypt were counted and percentage of goblet cells calculated.

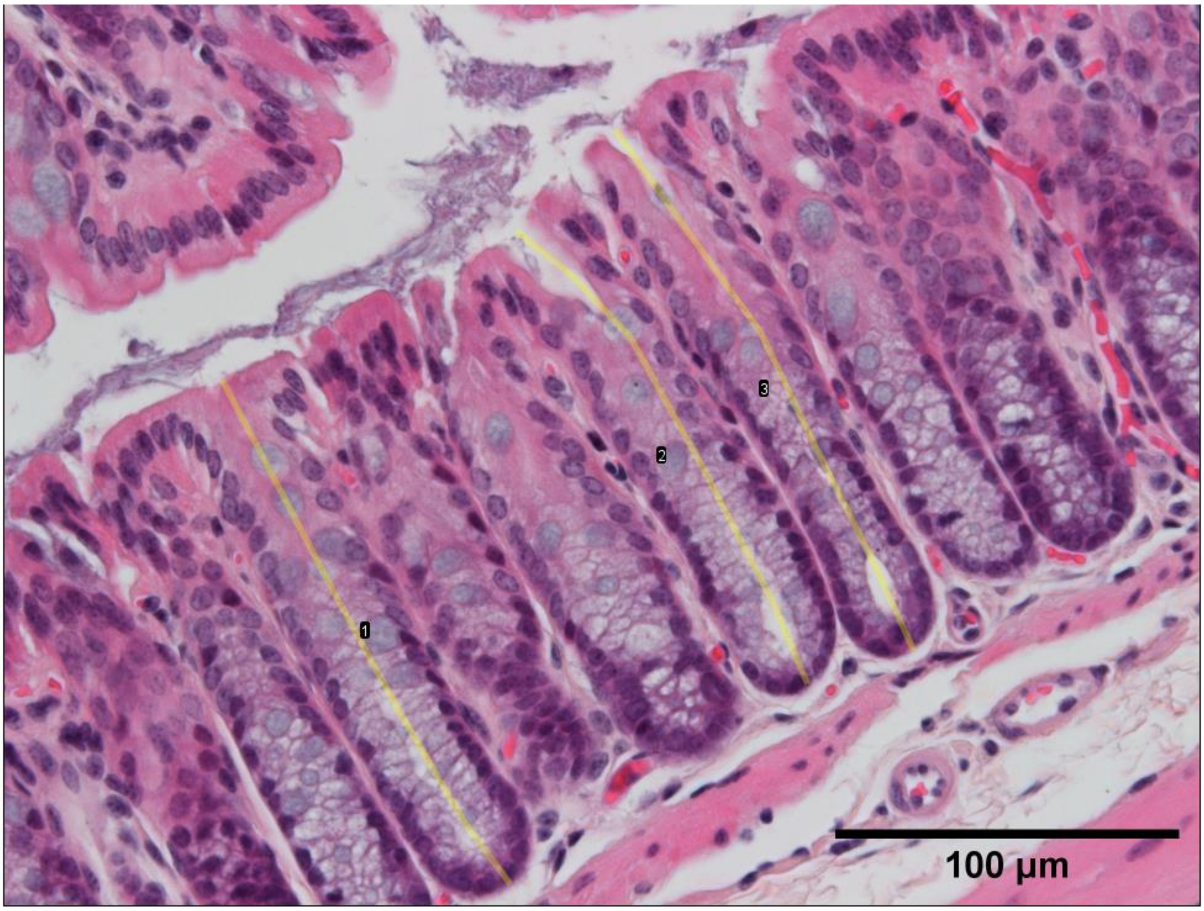

Supplementary Figure S6: Example of colon crypt length measurement. Yellow lines depict the measurement lines.

Gene expression experiments

For lysation 30 mg of each tissue were added to 30 mg *dia Zirconia Silica* beads (0.1 mm) and 600 µL RLT Buffer (Qiagen #79216) in 2 mL scMirco tubes. Next, tissues were lysed using *FastPrep* (6 m/sec for 40 sec) and samples were centrifugated at 13000 rpm for 5 minutes at 4 °C.

For DNase digestion, 1 µg RNA was mixed with 1 µL DNase, 1 µL 10x Reaction Buffer containing $MgCl_2$ and DEPC water to 10 µL. Samples were placed in RNase free tubes for 30 minutes at 37 °C, followed by addition of 1 µL of 50 mM EDTA at 65 °C for 10 minutes.

Details for PCR reaction were as follows: Each PCR amplification reaction (20 µL total volume) containing 50 ng of synthesized cDNA template and 0.4 to 0.8 µM of forward and reverse primers

(**Supplementary Table 4**). The thermal cycle started with hot start polymerase activation at 95 °C (2 min), followed by 40 cycles of denaturation at 95 °C (15 sec) and annealing at 60 °C (1 min) for the most primer pairs. For some primers, the annealing temperature and time were adjusted, or elongation was performed (72 °C, 30 sec). Adjustments to the cycling program are listed in **Supplementary Table 4**.

Intestinal microbiome analysis: - Detailed protocol for 16S rRNA gene sequencing

DNA extraction

Mouse feces was collected for microbiome analysis one day after feeding with the experimental diets started (= week 0) and after 36 days (= week 5). For one mouse of diet group 30S, no feces could be collected at week 0. Intestinal contents were collected during the dissection of the mice (= week 12) from the ileum, caecum and colon. All samples were flash frozen in liquid N_2 and stored at -80 °C. DNA was extracted from all samples using the NucleoSpin DNA Stool kit (Macherey-Nagel). Up to 200 mg of feces were transferred to the bead tubes, incubated with 850 µL lysis buffer at 70 °C for 5 min and then subjected to 40 s treatment on a FastPrep 24 machine (MP biomedicals) at 6.0 m/s, followed by washing and elution of DNA according to the kit manufacturer's protocol. Purity and concentration of the extracted DNA were checked on a microvolume photometric device.

16S rRNA gene sequencing

The variable region V4 of the prokaryotic 16S rRNA gene was amplified in a single PCR step with dual indexing primers as described by Kozich *et al.* [1] with modifications by Parada *et al.* [2] and Apprill *et al.* [3] (**Supplementary Table 5**). NEBNext® Ultra™ II Q5® Master Mix (New England Biolabs) was used in a 25 µL reaction mix with 1 ng template DNA, 0.5 µM of each PCR primer (forward and reverse), 0.2 mM dNTP mix, 12.5 µL Q5 Master Mix, filled up with HyPure water (Cytiva Europe GmbH). The PCR program included initial denaturation for 30 sec at 98 °C, 25 cycles of 10 s at 98 °C, 30 s at 55 °C and 20 s at 72 °C, followed by terminal elongation for 2 min at 72 °C. PCR products were purified using magnetic beads (Mag-Bind® RxnPure Plus, Omega) and their concentration was measured using a Quantus Fluorometer (Promega) with the QuantiFluor One dsDNA System (Promega), according to the manufacturer's recommendation. Negative control samples included amplification without template and using DNA purifications without sample as template (= blank control). The PCR products were pooled at equimolar concentration to 5 libraries that were sequenced on individual runs (some including libraries of other projects) using the Illumina MiSeq v3 kit following the manufacturer's instruction with modifications. To enable sequencing of the dual indexing primers, 3.4 µL of 100 µM custom sequencing primers (**Supplementary Table 5**) were added to the cartridge as described in the MiSeq WetLab SOP by Kozich *et al.* [4]. Detailed information on conditions of the 5 sequencing runs are listed in **Supplementary Table 6**.

Bioinformatic analysis

Bioinformatic analysis was performed in R version 4.1.0 [5]. Raw sequence data were trimmed to 200 (fwd) and 150 (rev) bases and reads with ambiguous base calls or an expected error rate > 2 after trimming were discarded. Remaining reads were error corrected, forward and reverse reads merged, and chimeric sequences removed using *dada2* version 1.22 [6] with the 'pseudo-pooling' option yielding 1,249 amplicon sequence variants (ASVs) of the V4 region. ASVs were classified using the RDP Naïve Bayesian Classifier algorithm as implemented in dada2 against the Silva database v138.1 [7]. Were possible by exact matching, ASVs were assigned species names using the *assignSpecies* function of *dada2*. After the preprocessing the data amounted to between 10,393 and 513,237 sequences per sample. Two ileum samples yielded very low read counts (< 500 sequences) and had to be excluded.The *phyloseq* package v1.38.0 [8] was used to determine alpha-diversity as richness (Chao1 index [9]) and

Shannon diversity [10], beta-diversity using the Jenson-Shannon-Divergence (JSD) metric and perform principal coordinate analyses. The functions *lmer* (mixed linear models, package *lmerTest* v3.1-3 [11]) and *adonis2* (permutational multivariate analysis of variance, PERMANOVA, package *vegan* v2.6-2 [12]) were used for statistical tests on group differences in alpha- and beta-diversity, respectively. PERMANOVA analysis used 9999 permutations. Correlations between abundance of microbial taxa and metabolite concentrations were calculated as Pearson's correlation coefficient using the function *corr.test* (package *psych* v2.2.5, [13]). The package *ALDEx2* v1.26.0 [14] was used for *centered log ratio* (*clr*) transformation of microbial abundances and test for group differences using *generalized linear models* (*glm*). Data were visualized using the *ggplot2* package v3.3.6 [15].

Detailed protocol for GC-Analysis of SCFA and BCFA in caecum content and feces

About 100 mg of caecum or feces were freeze-dried using an Alpha 1-2 freeze-drying manifold (Martin Christ), resulting in about 25 mg (range 5 to 70 mg) of dry material. 880 µL deionized water, 20 µL internal standard (ISTD, 4-ethylbutyric acid, 1 g/L) and 100 µL HCl (25 %) were successively added, leading to an end volume of 1000 µL. Samples were placed in an overhead mixer for 10 min, followed by vortexing for 10 min, and then centrifugation for 10 min at 16,100 rpm (23,100 g). The supernatant was transferred to another tube and 200 µL were transferred to conic GC vials. Samples were analyzed using a 6890 GC (Agilent), equipped with a split/splitless injector. A 0.5 µL sample was injected using a split ratio of 1:10 at 200 °C. Water/HCl and methanol were used as wash solvents. SCFAs were separated on a ZB-WAXplus column with the dimensions 30 m × 0.25 mm × 0.25 µm (Phenomenex). Hydrogen was used as carrier gas with a flow rate of 42 cm/sec. The following temperature program was used: 70 °C (0.3 min)/70 to 230 °C (21° C/min; 8.4 min)/230 (0.5 min). Individual SCFAs were detected by FID at 250 °C using helium as makeup gas. Compounds were quantified by internal calibration (7 levels). A volatile acid mix (10 mM) was complemented by additional acetic acid spike and those single FA which were not included in the commercial mixture (all standards from Sigma-Aldrich). Appropriate volumes of these stock solutions were mixed and further diluted to desired calibration levels by adding 20 µL ISTD, 100 µL HCl (25 %) and filling up to 1000 µL with deionized water. The calibration range was up to 1000 µM, and 6-fold higher for acetic acid (6250 µM). Freeze-dried matrix controls (2 levels, with and without spike) were used to monitor method performance.

Comparison between microbiota and SCFA/BCFA profiles

Associations between the caecal microbiota and SCFA/BCFA contents were calculated as correlations between relative abundances of taxa and concentrations (µmol/g) of metabolites. Taxa abundances were filtered to remove rare taxa with a maximum relative abundance of 1 % within the caecum samples. Correlations were calculated as Pearson's linear correlation coefficient using the *corr.test* function of R package *psych [13]*. Correlations were visualized as a heatmap with *heatmap.2*, package *gplots [16]*. Clusters of taxa that showed similar correlations with SCFA/BCFA profiles were calculated using hierarchical clustering (*hclust*, UPGMA method) of the euclidean distances, and dividing the resulting dendrogram into three clusters using *cutree* (k = 3). Relative abundances of the three clusters were calculated by merging all ASVs of the same cluster using the *merge_taxa* command of the *phyloseq* package.

References:

1. Kozich, J.J., et al., *Development of a dual-index sequencing strategy and curation pipeline for analyzing amplicon sequence data on the MiSeq Illumina sequencing platform.* Appl Environ Microbiol, 2013. **79**(17): p. 5112-20.
2. Parada, A.E., D.M. Needham, and J.A. Fuhrman, *Every base matters: assessing small subunit rRNA primers for marine microbiomes with mock communities, time series and global field samples.* Environ Microbiol, 2016. **18**(5): p. 1403-14.
3. Apprill, A., et al., *Minor revision to V4 region SSU rRNA 806R gene primer greatly increases detection of SAR11 bacterioplankton.* Aquatic Microbial Ecology, 2015. **75**(2): p. 129-137.
4. Kozich, J.J., et al. *MiSeq Wet Lab SOP*. 2013; Available from: https://github.com/SchlossLab/MiSeq_WetLab_SOP/blob/master/MiSeq_WetLab_SOP_v4.md.
5. R Core Team. *R: A Language and Environment for Statistical Computing*. 2019; Available from: https://www.R-project.org.
6. Callahan, B.J., et al., *DADA2: High-resolution sample inference from Illumina amplicon data.* Nat Methods, 2016. **13**(7): p. 581-3.
7. Quast, C., et al., *The SILVA ribosomal RNA gene database project: improved data processing and web-based tools.* Nucleic Acids Res, 2013. **41**(Database issue): p. D590-6.
8. McMurdie, P.J. and S. Holmes, *phyloseq: an R package for reproducible interactive analysis and graphics of microbiome census data.* PLoS One, 2013. **8**(4): p. e61217.
9. Chao, A., *Nonparametric-Estimation of the Number of Classes in a Population.* Scandinavian Journal of Statistics, 1984. **11**(4): p. 265-270.
10. Simpson, E.H., *Measurement of Diversity.* Nature, 1949. **163**: p. 688.
11. Kuznetsova, A., P.B. Brockhoff, and R.H.B. Christensen, *lmerTest Package: Tests in Linear Mixed Effects Models.* Journal of Statistical Software, 2017. **82**(13): p. 1-26.
12. Oksanen, J., et al. *vegan: Community Ecology Package (version 2.5-1)*. 2018; Available from: https://CRAN.R-project.org/package=vegan.
13. Revelle, W. *psych: Procedures for Psychological, Psychometric, and Personality Research*. 2022; Available from: https://CRAN.R-project.org/package=psych.
14. Fernandes, A.D., et al., *Unifying the analysis of high-throughput sequencing datasets: characterizing RNA-seq, 16S rRNA gene sequencing and selective growth experiments by compositional data analysis.* Microbiome, 2014. **2**: p. 15.
15. Wickham, H., *ggplot2: Elegant Graphics for Data Analysis*. 2009: Spinger-Verlag New York.
16. Warnes, G.R., et al. *Package 'gplots': Various programming tools for plotting data. Version 3.0.1.1*. 2016; Available from: https://cran.r-project.org/web/packages/gplots/index.html.
